# Melatonin analgesia is associated with improvement of the descending endogenous pain-modulating system in fibromyalgia: a phase II, randomized, double-dummy, controlled trial

**DOI:** 10.1186/2050-6511-15-40

**Published:** 2014-07-23

**Authors:** Simone Azevedo de Zanette, Rafael Vercelino, Gabriela Laste, Joanna Ripoll Rozisky, André Schwertner, Caroline Buzzatti Machado, Fernando Xavier, Izabel Cristina Custódio de Souza, Alicia Deitos, Iraci L S Torres, Wolnei Caumo

**Affiliations:** 1 Pain and Palliative Care Service at the Hospital de Clínicas de Porto Alegre (HCPA), Universidade Federal do Rio Grande do Sul (UFRGS), Porto Alegre, Brazil; 2 Pharmacology Department, Instituto de Ciências Básicas da Saúde, UFRGS, Porto Alegre, Brazil; 3 Post Graduate Program in Medical Sciences, School of Medicine, Universidade Federal do Rio Grande do Sul (UFRGS), Porto Alegre, Brazil; 4 Laboratory of Pain & Neuromodulation, Institution: Hospital de Clínicas de Porto Alegre at UFRGS, Rua Ramiro Barcelos, 2350 - CEP 90035-003 Bairro Rio Branco, Porto Alegre, Brazil

**Keywords:** Melatonin, Amitriptyline, CPM, BDNF, Clinical trial

## Abstract

**Background:**

Central disinhibition is a mechanism involved in the physiopathology of fibromyalgia. Melatonin can improve sleep quality, pain and pain threshold. We hypothesized that treatment with melatonin alone or in combination with amitriptyline would be superior to amitriptyline alone in modifying the endogenous pain-modulating system (PMS) as quantified by conditional pain modulation (CPM), and this change in CPM could be associated with serum brain-derived neurotrophic factor (BDNF). We also tested whether melatonin improves the clinical symptoms of pain, pain threshold and sleep quality.

**Methods:**

Sixty-three females, aged 18 to 65, were randomized to receive bedtime amitriptyline (25 mg) (n = 21), melatonin (10 mg) (n = 21) or melatonin (10 mg) + amitriptyline (25 mg) (n = 21) for a period of six weeks. The descending PMS was assessed with the CPM-TASK. It was assessed the pain score on the Visual Analog Scale (VAS 0-100 mm), the score on Fibromyalgia Impact Questionnaire (FIQ), heat pain threshold (HPT), sleep quality and BDNF serum. Delta values (post- minus pre-treatment) were used to compare the treatment effect. The outcomes variables were collected before, one and six weeks after initiating treatment.

**Results:**

Melatonin alone or in combination with amitriptyline reduced significantly pain on the VAS compared with amitriptyline alone (*P* < 0.01). The delta values on the VAS scores were-12.85 (19.93),-17.37 (18.69) and-20.93 (12.23) in the amitriptyline, melatonin and melatonin+amitriptyline groups, respectively. Melatonin alone and in combination increased the inhibitory PMS as assessed by the Numerical Pain Scale [NPS_(0-10)_] reduction during the CPM-TASK:-2.4 (2.04) melatonin + amitriptyline,-2.65 (1.68) melatonin, and-1.04 (2.06) amitriptyline, (P < 0.05). Melatonin + amitriptyline treated displayed better results than melatonin and amitriptyline alone in terms of FIQ and PPT improvement (P < 0.05, fort both).

**Conclusion:**

Melatonin increased the inhibitory endogenous pain-modulating system as assessed by the reduction on NPS_(0-10)_ during the CPM-TASK. Melatonin alone or associated with amitriptyline was better than amitriptyline alone in improving pain on the VAS, whereas its association with amitriptyline produced only marginal additional clinical effects on FIQ and PPT.

**Trial registration:**

Current controlled trail is registered at clinical trials.gov upon under number NCT02041455. Registered January 16, 2014.

## Background

Fibromyalgia (FM) is a syndrome characterized by chronic widespread musculoskeletal pain, hyperalgesia, allodynia, stiffness of the body, fatigue, sleep disorders, circadian rhythm disturbances, anxiety, depression and, commonly, a high level of catastrophizing related to pain [1,2]. A previous study demonstrated that patients with fibromyalgia have low melatonin secretion, which could explain the lack of restorative sleep [3], which is a predisposing factor in trigger point formation [4] and dysfunction of pain modulation mechanisms [3]. Melatonin can block the cycle of impaired sleep at night, fatigue during the day [5-7], and can induce circadian rhythm synchronization [8]. In addition, melatonin administration in mice has antidepressant effects [9] and, in humans, anxiolytic properties [10].

Melatonin’s effect on pain has been demonstrated in animals for inflammatory [8] and neuropathic pain [11-13], as well in acute [10,14] and chronic pain in humans [15,16]. In addition, there is some clinical evidence of melatonin’s effect on FM [5,6]. However, the various study results are not consistent, possibly because the dose used has been low (3-5 mg). In regard to the melatonin dose for pain, a recent randomized clinical trial (RCT) indicated that 10 mg at bedtime produced a large size effect for chronic pelvic pain [16]. In addition, melatonin reduced the BDNF serum level in patients with chronic pelvic pain induced by endometriosis [16].

Pain is a dynamic phenomenon resulting from the activity of both endogenous pain excitatory and inhibitory systems, including inhibitory conditioned pain modulation (ICPM) [17]. The efficacy of ICPM in FM has been related to sleep quality [18]. This relationship is supported on neurobiological grounds by common neurotransmitters involved in both sleep and ICPM, including noradrenalin (NA), serotonin (5-HT) and dopamine (DA) [19-22]. Thus, it is important to investigate the therapeutic effect of drugs such as melatonin that present multifaceted mechanisms that may interfere in the peripheral and central pain mechanisms. The rationale that supports this hypothesis consists of evidence that, in long-term chronic pain situations, there is a loss of inhibitory system function, as demonstrated in FM by the pain threshold and conditioned pain modulation (CPM) [23]. Accordingly, in a recent study, we demonstrated that long-term musculoskeletal pain occurs with excessive cortical facilitation (a lack of inhibition), which is associated with lower pain threshold and higher levels of catastrophizing thinking related to pain [24]. In addition, numerous pre-clinical studies have demonstrated that ICPM depends on the recruitment of endogenous opioids in the periaqueductal gray, which trigger the release of 5-HT from neurons localized in the raphe nuclei (medulla), which, in turn, dampens nociceptive afferents at the dorsal horn of the spinal cord [25]. Noradrenergic projections from the locus coeruleus produce similar effects [25]. Together, this evidence justifies assessing the effect of melatonin in the descending modulatory pain systems, alone or combined with classic therapeutic agents such as amitriptyline, as it has been demonstrated that melatonin increases the pain threshold in healthy subjects [26], improves sleep quality [15] and modulates systems involved in pain, such as the GABAergic and opioidergic systems [12,27,28].

Taking all of this information into account, we hypothesized that melatonin treatment alone or in combination with amitriptyline is better than amitriptyline alone at modifying the endogenous pain-modulating system. Thus, to prove our hypothesis, in this study, we quantified the conditioned pain modulation (CPM)-TASK, as well BDNF serum levels in FM patients who received melatonin treatment alone or in combination with amitriptyline. We also tested whether melatonin improved clinical symptoms such as pain, pain threshold (PPT) and sleep quality related to fibromyalgia.

## Methods

**Figure 1:**
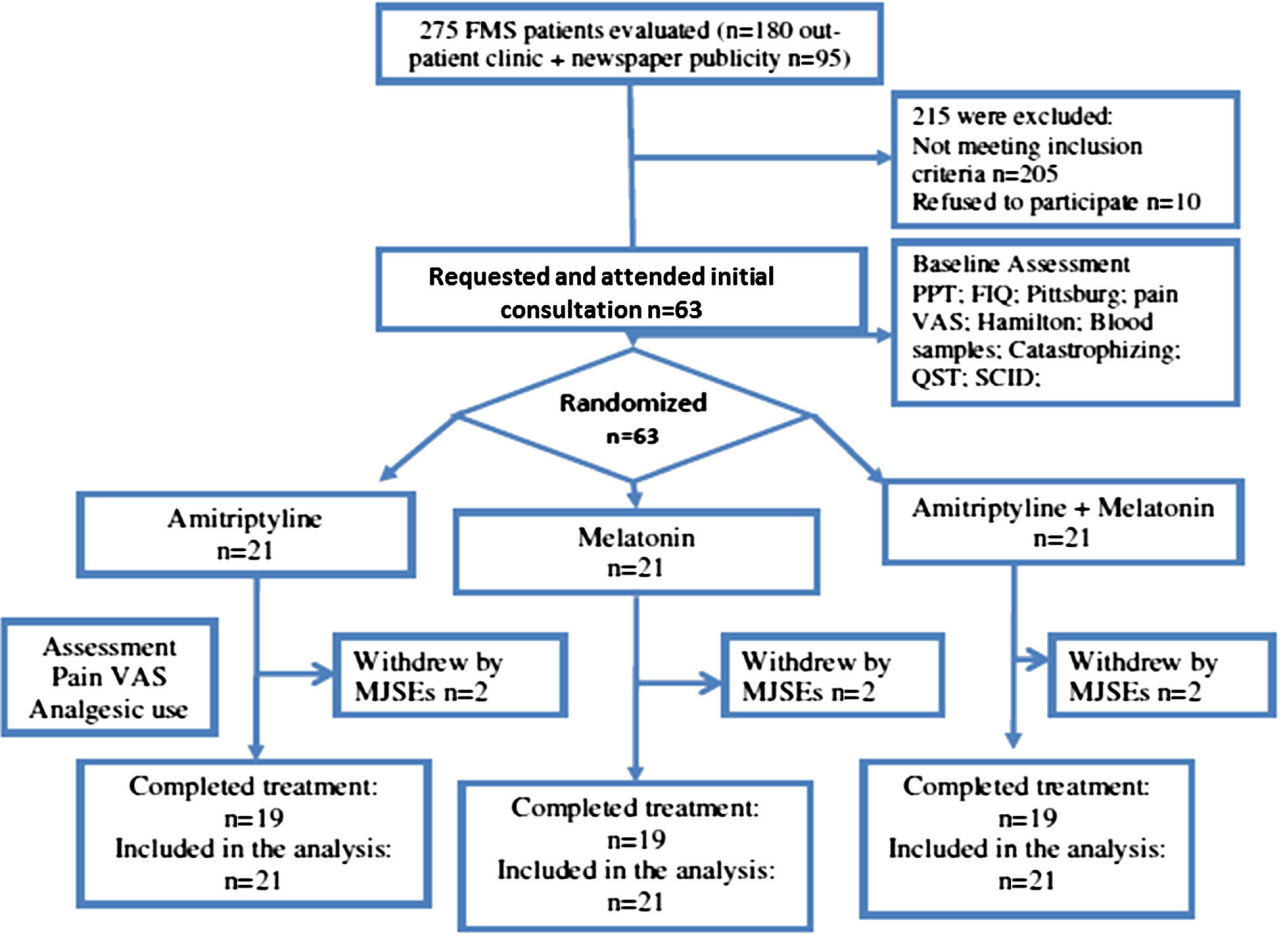
**Flow chart showing recruitment and progress through the study.** FMS: fibromyalgia syndrome; FIQ: fibromyalgia impact questionnaire; PPT: pain pressure threshold; QST: quantitative sensory testing; SCID: Structured Clinical Interview for DSM-IV; pain VAS: visual analog scale of pain; major side effects (MJSE) (severe dizziness, vivid nightmares, crippling drowsiness, severe headache, behavioral changes, and pain worsening).

The Methods and Results sections are reported according to the CONSORT guidelines [29]. Figure 1 shows the flow chart of the study.

### Design overview, setting, and participants

All patients provided written informed consent before participating in this randomized, double-blind, double-dummy, three-group parallel, clinical trial, which was approved by the Research Ethics Committee at the Hospital de Clínicas de Porto Alegre (HCPA) (Institutional Review Board IRB 0000921) in accordance with the Declaration of Helsinki (Resolution 196/96 of the National Health Council). We included 63 adult patients aged 18-years-old or older from among the pain and physiatrist clinical outpatients of the HCPA and via newspaper publicity. Patients with FM were enrolled according to American College of Rheumatology criteria [30]. To be eligible, patients had to be refractory to their current treatment. Patients were required to have a score of at least 50 mm on the 0-100 mm visual analogue scale (VAS, which 0 means “no pain” and 100 means “worst possible pain”) [31] during the baseline week preceding randomization and to have completed at least four pain diaries out of seven. Patients were allowed to remain on analgesic medications, including drugs for which they were refractory, and these medications could not be adjusted during the study. At screening, all patients underwent physical examination by a pain specialist and a psychiatric interview with a psychiatrist. Patients could enroll with or without a history of major depressive disorder; however, it could not be the main reason for their functional impairment or study enrollment. Subjects were recruited solely for fibromyalgia pain. Patients were excluded if evidence was found of inflammatory rheumatic disease, autoimmune disease or other painful disorders that might confound the assessment of fibromyalgia pain or a history of substance abuse. Patients who were pregnant, breast-feeding or had a history of neurologic or oncologic disease, ischemic heart disease, kidney or hepatic insufficiency were also excluded.

### Sample size justification

The number of patients in each study group was determined by previous clinical trials [32]. An *a priori* estimate indicated that a total sample size of 57 patients divided into three balanced treatment groups (n = 19) was needed to detect a 1.4-cm reduction (average standard deviation 1.2 cm) in pain intensity associated with melatonin or placebo at power and α levels of 0.8 and 0.01, respectively [33]; such a reduction would be clinically relevant and comparable to other pharmacological interventions. To account for multiple outcomes and attrition, we increased the sample size to 21 patients per group. For these calculations, we assumed that this remission was clinically relevant.

### Randomization and masking

The participants were randomized into one of three groups: amitriptyline (+placebo), melatonin (+placebo), and amitriptyline + melatonin. Before the recruitment phase, envelopes containing the protocol materials were prepared. Each envelope was sealed and numbered sequentially and contained an allocated treatment. After the participant consented to participate in the trial, in the sequence, the nurse, who administered the medications, opened the envelope. During the entire protocol timeline, two investigators who were not involved in patient evaluations were responsible for the blinding and randomization procedures. Other individuals who were involved in patient care were unaware of the treatment group to which the patients belonged.

### Interventions

Over a six-week period (42 days), the following oral medications were taken at bedtime by the three groups: melatonin (10 mg) tablets + placebo (Sigma Chemical, Germany, provided batch-by-batch certificates of analysis authenticating the purity of each batch), amitriptyline (25 mg) + placebo or amitriptyline (25 mg) + melatonin (10 mg) with identical characteristics. The capsules were manufactured in such a way that the placebo and active treatment had the same size, color, smell and flavor. To measure adherence to medication use, we employed the following strategies: ***i***) a researcher counted the number of tablets consumed weekly during the study period; ***ii***) the patients were asked to record a diary entry if they failed to use the medication; ***iii***) eligible patients were strongly encouraged to remain on the medication throughout the six weeks, during which time they visited the clinical center the third week after beginning the treatment. Regardless of their decision to continue or discontinue medication at this stage, the patients continued to be assessed during the study period.

### Supplementary analgesic use

All of the patients were permitted to use supplementary analgesic medication (acetaminophen, ibuprofen, codeine or tramadol) to relieve their pain if necessary. Patients were allowed to take 750 mg of acetaminophen up to four times per day (QID) and 200 mg of ibuprofen at maximum QID as a rescue analgesic. If their pain persisted, patients could use Dorflex® (Sanofi Aventis, São Paulo, Brazil; 35 mg of orfenadrine citrate combined with 300 mg of dypirone and 50 mg of caffeine). If their pain persisted, patients were permitted to use 60 mg of codeine up to QID or tramadol three times per day (TID). These medications could be used a maximum of four times a day. The patients were asked to record their analgesic intake during the treatment period in their diaries, and these diaries were reviewed at the end of the treatment section. The total analgesic dose taken during the last week of treatment was considered for the analysis.

### Instruments and assessments

All of the psychological tests used in this study have been validated for the Brazilian population [34,35]. Two independent medical examiners that were blind to the group assignments were trained to administer the pain scales and conduct the psychological tests. The baseline depressive symptoms of the patients were assessed using the Hamilton Depression Scale [35], and sleep quality was assessed using the Pittsburgh Sleep Quality Index [36]. Psychiatric disorders were evaluated with the Structured Clinical Interview for DSM-IV Axis I Disorders (SCID-I) [37]. To assess catastrophic thinking due to chronic pain, we used the B-PCS [38]. Demographic data and medical comorbidities were assessed using a standardized questionnaire; patients were asked about any changes that occurred during treatment, such as changes in mood, sleepiness, dizziness, headaches or allergic reactions.

### Outcomes

The primary outcomes were pain score diaries on the Visual Analog Scale (VAS) [global pain in the last 24 hours] obtained during the last week of treatment and pain reduction on the Numerical Pain Scale [NPS_(0-10)_] during the CPM-TASK. The secondary outcomes were the amount of analgesics used in the last week of treatment, the score on Fibromyalgia Impact Questionnaire (FIQ), Pressure Pain Thresholds (PPT), sleep quality and BDNF serum levels. The outcomes are described below.

#### Assessment of pain and sleep quality

a) The pain intensity was measured with a 100-mm VAS. The VAS scores ranged from no pain (zero) to worst possible pain (100 mm). The time of the worst pain during the last 24 h was recorded daily in the patients’ diaries obtained at baseline during the seven days before beginning the treatment and the last week of the treatment period. They were asked to answer the following question using the pain VAS: ***i***) considering your pain, how intense was your worst pain during the last 24 hours? Diary entries recorded analgesic intake (acetaminophen, non-steroidal anti-inflammatory drugs (NSAIDs) or opioids). The total analgesic dose taken during the last week of treatment was considered for analysis.

b) The quality of life of the patients in this study was evaluated using the Fibromyalgia Impact Questionnaire (FIQ), a disease-specific questionnaire initially proposed by Burckhardt et al. [39] for the evaluation of quality of life in patients with fibromyalgia. It was validated for use in the Brazilian population by Marques et al. [40]. This questionnaire is composed of 10 domains, the first consisting of 10 sub-items or questions, and the other nine of only one question each. The first domain contains questions concerning the capacity of the patient to perform certain routine activities. Responses range from 0, always able to perform the activity, to 3, never able to perform the activity. Item two refers to the number of days during that the patient felt well in the previous week, and item three refers to the number of days that the patient was unable to go to work because of the disease. Possible answers range from 0 to 7 for each item or domain. For domains 4-10, the scores range from 0 to 10 in each. These final seven items are designed to collect data on the patient’s capacity to work and their perceptions of pain, fatigue, morning stiffness, mood, anxiety and depression. The data from the FIQ are arranged so that no more than 10 points can be scored for any single item. Items 2 and 3 are considered inversely proportional; therefore, the maximum possible score in this questionnaire will generally be 100.

c) Sleep quality during the study period was assessed using the Pittsburgh Sleep Quality Index [36].

### Assessment of PPT and conditioned pain modulation

a) Pressure pain thresholds (PPT alone): Prior to the test trial, the patient learned to differentiate the perception of pressure versus the perception of the onset of pain. The patient was instructed to verbally report the perception of pain onset. The investigator who assessed pain threshold levels was trained, blinded to the intervention and unable to view the display of pressure intensities. An experienced rehabilitation physician (SAZ) systematically evaluated superficial and deep hyperalgesia by assessing PPT using an algometer [41]. The device had a 1-cm^2^ hard-rubber probe, which was applied over eleven predefined different areas to define the PPT. These areas are among the nineteen areas corresponding to the diagnosis of FM according to the American College of Rheumatology criteria [30]. We determined the individual’s PPT using the area that presented the lowest PPT. The average values of PPT in kgf/cm^2^ (lb/cm^2^) for three successive readings taken at intervals of 3-5 min were used as the outcomes.

b) To test CPM (the term CPM rather than diffuse noxious inhibitory control [DNIC] is chosen based on the recent recommendations of Yarnitsky et al. [17]), we used the protocol of Tousignant-Laflamme [42] and consulted the guidelines for the cold-heat task (CPM-TASK) as an experimental pain stimulus [43]. The CPM-TASK activates the diffuse noxious inhibitory control-like effect (CPM), as it is a strong nociceptive stimulus that takes place over a lengthy span of time [44] and is applied over a large body surface area [45]. Thus, the CPM-TASK allows us to modify the endogenous pain-modulating system. To quantify CPM, we evaluated the pain intensity of three heat pain (HPT) test stimuli separated by a CPM-TASK. Even if the HPT may lead to both habituation and sensitization according to the dual process theory, the cold water zero is a reliable stimulus to induce CPM [42].

c) CPM-Task: The cold-heat task was used as a conditioning stimulus to elicit a strong and prolonged pain sensation to trigger CPM. The CPM-TASK consisted of immersing the non-dominant hand cold water (zero to 1°C) for 1 minute. During the last 30 seconds of cold-water immersion, the HPT procedure was administered over the right forearm (dominant forearm). The temperature was held constant during the experiment for each subject. The mean temperature eliciting pain ratings of 0/10 on the Numerical Pain Scale [(NPS)_0-10_] (HPT) was used for the HPT. After a short break, the first HPT (Pain baseline, HPT0) was applied at the extensor carpi radialis longus muscle (forearm) of the dominant forearm. Following the first HPT (HPT0), the CPM-TASK was used to trigger CPM. One minute after the CPM-TASK, we applied the second HPT (HPT1). We quantified the amount of CPM by subtracting the mean pain rating of the second HPT after the CPM-TASK (HPT1) from the first HPT before the CPM-TASK (HPT1) [46].

d) Laboratory outcomes included serum levels of BDNF. Samples of blood were collected at two time points: at baseline and at the end of treatment. The blood samples were centrifuged in plastic tubes for 10 min at 4500 × g at 4°C, and serum was stored at-80 °C for assay. Serum BDNF was determined by the Enzyme-Linked Immunosorbent Assay (ELISA) using a ChemiKine BDNF Sandwich ELISA Kit, CYT306 (Chemicon/Millipore, Billerica, MA, USA). The lower detection limit of the kit is 7.8 pg/mL of BDNF.

### Statistical Analysis

The differences between the groups at baseline were examined by analysis of variance (ANOVA) for parametric variables with normal distribution or the Kruskal-Wallis test, and categorical variables were examined using chi-square or Fisher’s exact tests given that our main independent outcome (intervention) was also categorical.

The results were evaluated using the absolute mean variation of pain measurements quality and BDNF were evaluating using delta values (post-treatment minus pre-treatment). Several outcomes of pain measurements (VAS, FIQ, PPT, number of tender points and PPT) did not present normal distribution. Linear mixed models were used to compare outcomes within subjects and between subjects in which the independent variable was the treatment (amitriptyline, melatonin and melatonin + amitriptyline) with Bonferroni’s Multiple Comparison Test. The reduction on the NPS_(0-10)_ induced by the CPM-TASK was adjusted for the baseline HPT and serum BDNF.

To identify possible predictors associated with the change on the NPS_(0-10)_ during the CPM-TASK, we fitted a multiple linear regression model, using the stepwise enter method. The variables included in the model were as follows: B-PCS score, HPT0, and FIQ score obtained before treatment. In addition, the number of analgesics used in the last week of treatment was included. Finally, an exploratory analysis using the Spearman correlation coefficient (*r*_*s*_) was performed to understand the reverse effect of the interaction of HPT0 and BDNF [each alone presented a negative correlation with NPS_(0-10)_ during the CPM-TASK] that becomes positive. Within groups, the standardized mean difference (SMD) was computed in terms of the ratio between the mean change and the pool of baseline standard deviation (SD). The SMD was interpreted as follows: small, 0.20 to 0.4; moderate, 0.50-0.70 and large, 0.80 or higher, with respective confidence interval (CI) [47]. All of the analyses were performed assuming intention-to-treat and thus included all of the randomized subjects for whom there were observations in the study outcomes. The analyses were performed with SPSS version 18.0 (SPSS, Chicago, IL).

## Results

### Patient characteristics

The clinical and demographic characteristics of the patients are shown in Table 1. Twenty-one patients were allocated to the amitriptyline group, 21 were allocated to the melatonin group, and 21 patients were allocated to the melatonin + amitriptyline group. Fifty-seven patients completed the study; two patients in the melatonin + amitriptyline group and two patients in both the amitriptyline and melatonin group withdrew because of treatment side effects. Baseline characteristics are presented in Table 1. The randomization produced balance groups for most part of characteristics, but, there was observed imbalance between groups in pain on the VAS, FIQ and PPT (Table 1). Regarding the side effects, in the amitriptyline group, 38.09% (8/21) of patients presented minor side effects (MSE) (nausea, mild dizziness, weight gain, dry mouth, and mild headache) and 23.8% (5/21) of patients presented major side effects (MJSE) (severe dizziness, vivid nightmares, crippling drowsiness, severe headache, behavioral changes, and pain worsening), with two withdraw. In the melatonin group, 23.8% (5/21) of patients presented MSE and 23.8% (5/21) of MJSE, with two withdrawals. The association of melatonin + amitriptyline resulted in 14% (3/21) of patients experiencing MSE, whereas 28.57% (6/21) of patients presented MJSE with two withdrawals. The comparisons in the incidence of MSE between the amitriptyline and the melatonin + amitriptyline group was statistically significant (P < 0.001). However, neither the incidence of MSE nor the incidence of MJSE was significant when the groups were compared (P > 0.05, for all comparisons).

**Table 1:** Epidemiological and clinical characteristics at baseline, according to the treatment group, values are given as the mean (SD) or frequency (n=63)

	**Amitriptyline (n = 21)**	**Melatonin (n = 21)**	**Amitriptyline + melatonin (n = 21)**	**P value**
Age (years)	49.80 ± (8.91)	47.40 ± (7.84)	49.72 ± (7.24)	0.59
Body index	27.65 ± (3.91)	27.18 ± (4.04)	27.58 ± (4.62)	0.60
Education (years)	10.95 ± (5.09)	11.30 ± (3.76)	8.22 + (5.6)	0.39
Smoking (n/%)	2 (9,5)	2 (9,5)	1 (5)	0.83
Clinical Comorbidity Yes/No	10 (47,6)	9 (42,9)	13 (65)	0.33
Hypertension (n/%)	5 (23,8)	5 (23,8)	8 (40)	
Hypothyroidism (n/%)	3 (14,3)	1 (4,8)	7 (35)	
Asthma (n/%)	1 (4,8)	1 (4,8)	2 (10)	
Other (n/%)	3 (15)	0 (0)	1 (4,8)	
Global pain on visual analogue scale^†^	62.88 ± (14.26)^a^	64.90 ± (15.44)^a^	69.57 ± (10.94)^b^	0.03
FIQ^†^	53.78 ± (12.83 )^a^	64.87 ± (12.83)^b^	65.15 ± (9.94)^b^	0.005
Pain catastrophizing scale for the Brazilian population (B-PCS)^†^				
	25.90 ± (10.38)	32.90 ± (12.28)	25.90 ± (10.38)	0.07
Pittsburgh Sleep Questionnaire	19.31 ± (6.58)	22.67 ± (7.69)	24.28 ± (7.79)	0.16
Hamilton Depression Scale^†^	17.61 ± (6.37)	21.70 ± (5.88)	17.61 ± (6.33)	0.05
Pain pressure threshold^†^	2.17 ± (0.21)^a^	1.99 ± (0.16)^b^	2.05 ± (0.24)^a^	0.04
Brain-derived neurotrophic factor (BDNF)^€^				
Mean (SD)	48.28 ± (24.00)	52.54 ± (23.87)	48.28 ± (27.34)	
Median [interquartile (IQ) 25;75]	45.51 (12.8;101.51)	54.78 (20.66;97.85	37.51 (18.75;92.3)	0.79
Psychiatric disease (SCID-I)	6 (76%)	15 (71%)	13 (65)	0.45
Depression	8 (38%)	1 (62%)	11 (55%)	
Anxiety	11(52%)	12 (57%)	5 (25%)	
Analgesic used weekly in last 3 months				
Median (Q_25-75_)^€^	6 (3;28)	7 (2;26)	7 (3;29	0.91
Analgesic use: days/week in last 3 months^¥^				
(<4 = no, ≥4 = times)	20 (95%)	20 (95%)	17 (85%)	0.43
A e aminophen/Dipirone	19 (90,5%)	16 (76,2%)	15 (75%)	
NSAID	7 (33,3%)	10 (47,6%)	9 (45%)	
Opioid	1 (4,8%)	0	0	
Active use of central nervous system active medication^¥^				
Yes/No	14 (66,7%)	16 (76%)	14 (70%)	0.16
Antidepressant (n/%)	14 (66,7%)	15 (71%)	13 (65%)	
Anticonvulsant (n/%)	2 (9,5%)	2 (9,5%)	1 (5)	
Benzodiazepine (n/%)	0	5 (23,8%)	2 (10%)	

Different superscripts (a and b) indicate significant differences among treatment groups according to the Bonferroni test.

Structured Clinical Interview for DSM-IV Axis I Disorders (SCID-I).

^€^Kruskal-Wallis Test.

^¥^Chi-Square or Fisher’s test.

^†^ANOVA.

### Analysis of the treatment effect on the main outcomes: descending modulatory system and pain on the VAS

**Figure 2:**
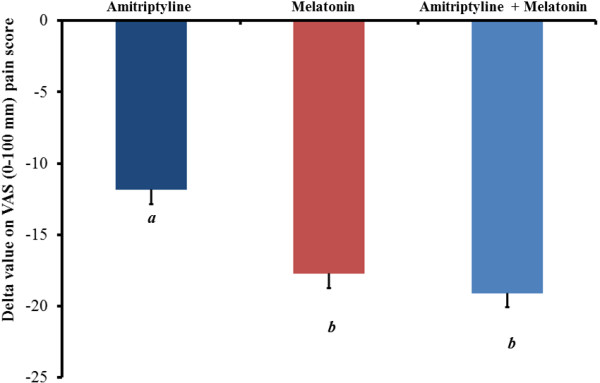
**Mean pain levels as Delta value (scores on VAS (0-100 mm) in last week of treatment minus scores one week pretreatment) in the three experimental groups.** The error bars indicate the standard error of the mean. A letter *b* indicates a significant difference between the melatonin group and melatonin + amitriptyline groups compared with the amitriptyline group (P < 0.05). All comparisons were performed using a mixed analysis of variance (ANOVA) model, followed by the Bonferroni correction for *post hoc* multiple comparisons.

Patients receiving melatonin alone or in combination with amitriptyline had significantly lower pain on the VAS than those receiving amitriptyline alone (*P* < 0.01) (Figure 2). A mixed model analysis revealed that the increase of one supplementary analgesic dose was associated with a 5.1% increase in the adjusted VAS pain change from baseline (t = 5.87; *P* < 0.001).

The change within the group was significant in all treatment groups (*P* < 0.001, for all comparisons) (Table 2). The cumulative mean (SD) on VAS one week pretreatment *vs.* the pain scores in the last week of treatment in the amitriptyline group was 62.87 (14.26) vs. 50.02 (25.60). For the melatonin group, these values were 64.90 (15.43) vs. 47.53 (21.96), respectively, and for the melatonin + amitriptyline group, the values were 69.57 (9.09) vs. 48.64 (15.38)], respectively. The effect size assessed by SDM [confidence interval (CI) 95%] within group in the amitriptyline group was 0.99 (CI 95%, 0.68-1.30), whereas in the melatonin group, it was 1.29 (CI 95%, 0.98-1.58), and in the melatonin + amitriptyline group, it was 1.47 (CI 95% 1.14-1.79) (Table 2).

**Table 2:** Multivariate linear regression of the interaction between the change in NPS (0-10) during the CPM-TASK by the treatment group considering the BDNF and pain thresholds (n = 63)

**Parameters**		***Β***	**t**	**P**	**95% CI**
**Dependent variable:** CPM-TASK					
		9.0	1.14	0.01	(4.33 to 32.35)
Treatment group					
	Melatonin + Amitriptyline	-1.27	-1.72	0.09	(-2.76 to 0.21)
	Melatonin	-1.75	-2.5	0.01	(-3.18 to-0.31)
	Amitriptyline	0^b(reference)^			
BDNF (ng/mL)		-0.28	-2.23	0.01	(-0.53 to-0.04)
Heat pain threshold		-8.06	-2.49	0.01	(-14.56 to-1.55)
Interaction					
	Serum BDNF (ng/mL) vs. PPT Pain pressure threshold (Kgf/cm^2^)	0.12	2.07	0.04	(0.04 to 0.24)

^b^indicates the reference category used to do the comparisons.

**Figure 3:**
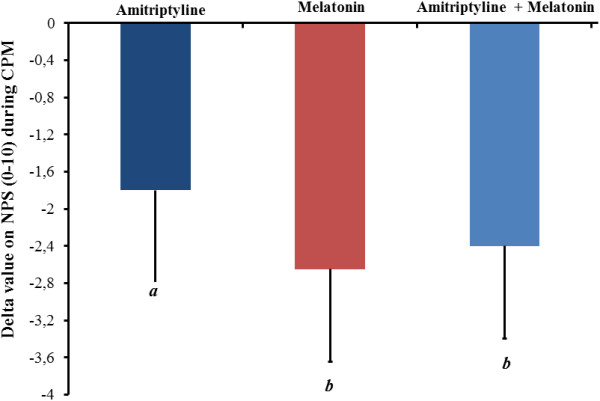
**Delta value on the pain NPS**_**(0-10) **_**during the CPM-TASK.** The error bars indicate the standard error of the mean. A letter *b* indicates a significant difference between the melatonin + and melatonin + amitriptyline compared with the amitriptyline group. All comparisons were performed using a mixed analysis of variance (ANOVA) model, followed by the Bonferroni correction for *post hoc* multiple comparisons.

The descending modulatory system function was assessed using the CPM-TASK. It was observed that in the overall treatment group, there was a reduction in pain scores during the CPM-TASK: melatonin + amitriptyline [HPT0 6.89 (1.92) vs. 4.49 (2.17) HPT1], melatonin [PPT0 7.52 (1.39) vs. 4.87 (1.97) HPT1] and amitriptyline [HPT0 6.07 (1.95) vs. 5.03 (2.17) HPT1]. The effect of treatment on CPM is presented in Figure 3. The CPM-TASK induced a reduction in pain in the melatonin group and melatonin + amitriptyline group that was significantly higher than in the amitriptyline group. However, it was observed that there were patients in all treatment groups that reported increased pain intensity (conditioned pain modulation-CPM) between the HPT0 pain measures (test stimulus) and HPT1 after the cold pressor task (CPM-TASK, conditioning stimulus). This characterizes a summation effect, with an incidence of 15.8% (3/21) in the melatonin group, 36.8% (8/21) in melatonin + amitriptyline group and 42% (9/21) in the amitriptyline-treated group. These results reveal that the descending modulatory system lost its inhibitory function as the heterotopy stimulus induced an increase in pain, when a reduction would be expected [42].

To understand this result, we performed an exploratory analysis to identify possible factors associated with this summation effect on pain score during the CPM-TASK. To identify possible predictors, a multiple linear regression model was constructed using the stepwise method. The variables included in the model were the B-PCS score, HPT0, and FIQ score obtained before treatment. In addition, the number of analgesics used in the last week of treatment was included. Accordingly, the HPT was the only variable retained in the model, that is, associated with pain changes on NPS (0 – 10) during the CPM-TASK [(r-square =-0.21), standard β coefficient for the HPT =-5.99, *P* < 0.05)].

Thus, a mixed model was constructed to assess the effect of treatment groups and its relationship with the serum BDNF adjusted by the HPT. It was observed that the HPT and serum BDNF were inversely correlated with the change on the NPS_(0-10)_ during the CPM-TASK (Table 3). However, when the interaction between HPT and BDNF was analyzed, this effect changed direction (i.e., it became positively associated with the change on the NPS_(0-10)_ during the CPM-TASK) (Table 3). To understand this result, we performed a simple correlation between serum BDNF and HPT, and the Spearman coefficient (*r*_*s*_) was-0.35 (P = 0.02). That is, when the HPT0 is low (higher the pain), the serum level of BDNF is high and vice versa. Thus, it is plausible to suppose that the effect of this interaction (HPT*serum BDNF) on pain score during the CPM-TASK was negatively correlated when included in the model as an isolated factor; it becomes in a factor correlated positively with the change reduction during the CPM-TASK (Table 3).

**Table 3:** The mean delta score [standard deviation (SD)] (post-treatment values minus pre-treatment values) of pain measures (FIQ, PPT, analgesic consumption, tender points) and sleep quality (n = 63)

**2A. Secondary outcomes**					
**Treatment**	**Mean (SD)**	**Mean difference (SD)**	**Median of the difference (Quartile 75;25)**	**P Value**^**†**^	**SDM CI 95%**
**Fibromyalgia Impact Questionnaire (FIQ) score**					
Amitriptyline (n = 21)	41.16 (14.61) vs. 53.78 (12.83 )	-12.19 (16.27)	-13.78 (-24.39,-4.95)^**b^	0.04	0.88 (0.43-1.33)
Melatonin (n = 21)	46.42 (16.18) vs. 64.87 (12.83)	-17.73 (13.0)	-16.30 (-22.29,-12.94)^**b^		1.28 (0.83-1.74)
Amitriptyline + melatonin (n = 21)	40.89 (10.94) vs. 65.15 (9.94)	-24.65 (12.14)	-26.41 (-32.59,-14.50)^**a^		1.79 (1.29-2.28)
**b. Mean pressure pain threshold (PPT) in (kg/cm2/second)**					
Amitriptyline (n = 21)	2.34 (0.45) vs. 2.05 (0.24)	0.29 (0.31)	0.2 (0.1, 0.5)**^b^	0.03	0.69 (0.34-1.04)
Melatonin (n = 21)	2.47 (0.33) vs. 1.99 (0.16)	0.47 (0.34)	0.4 (0.23, 0.55)**^b^		1.13 (0.79-1.47)
Amitriptyline + melatonin (n = 21)	2.70 (0.23) vs. 2.17(0.21)	0.54 (0.60)	0.6 (0.5, 0.7)**^a^		1.27 (0.9-1.64)
**Analgesic doses (mean during the last week of treatment)**^**†**^					
Amitriptyline (n = 21)	1.35 (1.2) vs. 2.07 (1.37)	-0.72 (1.40)	-0.22 (-0.82, 0.54)*	0.98	1.03 (0.33-1.67)
Melatonin (n = 21)	1.33 (1.29) vs. 2.16 (1.20)	-0.79 (1.52)	-0.14 (-1, 0.57)*		1.09 (0.51-1.66)
Amitriptyline + melatonin (n = 21)	1.04 (0.92) vs. 2.10 (1.03)	-1.1 (1.14)	-0.35 (-0.74, 0.47)*		1.33 (0.77-1.89)
**Number of tender points**					
Amitriptyline (n = 21)	10.62 (3.36) vs. 14.10 (2.27)	-3.45 (0.84)	- 4 (-5, 1.25)**	0.89	1.99 (1.35-2.63)
Melatonin (n = 21)	10.95 (2.94) vs. 14.71 (1.70)	- 3.75 (2.46)	- 4 (-6.5,-2.25)**		2.17 (1.53-2.80)
Amitriptyline + melatonin (n = 21)	10.29 (3.15) vs. 14.61 (2.32)	- 4.18 (1.91)	- 4 (-5,-3)**		2.41 (1.72-3.10)
**Pittsburgh Sleep Questionnaire**					
Amitriptyline (n = 21)	11.84 (5.82) vs. 19.31 (6.58)	-7.47 (7.34)	-7 (-11,-4)*	0.94	1.07 (0.51-1.63)
Melatonin (n = 21)	16.68 (8.38) vs. 22.67 (7.69)	-6.42 (6.53)	-5 (-11,-2)*		0.9 (0.38-1.44)
Amitriptyline + melatonin (n = 21)	16.11 (8.40) vs. 24.28 (7.79)	-7.58 (1.91)	-8 (-12,-1.5)*		1.06 (0.52-1.59)

*P < 0.01, **P < 0.0001 Comparisons using Mixed ANOVA model.

^†^Mixed ANOVA model. Mean difference of group. Different superscripts (a, b, and c) indicate significant differences among treatment groups according to the Bonferroni test.

Standardized mean difference (SMD) [(pre minus post)/pool baseline standard deviation] with confidence interval (CI) 95%. The size effect was interpreted as follows: small, 0.20 to 0.40; moderate, 0.50-0.70; and large, 0.80 or higher.

### Analysis of the efficacy on secondary outcomes: the Delta of pain scores for FIQ score, PPT, tender points, analgesic use, sleep quality and BDNF

The between-group changes in pain, pain threshold, analgesic consumption and sleep quality are shown in Table 3. The post hoc analysis indicated significant differences between the amitriptyline + melatonin group and the melatonin group from the amitriptyline group in terms of the FIQ score and PPT. No significant difference was observed between groups in the numbers of analgesic used in the last week of treatment, sleep quality and number of tender points. We observed a large effect size within groups considering the change pre- to post-treatment in pain measures, number of tender points and sleep quality (Table 3).

**Figure 4:**
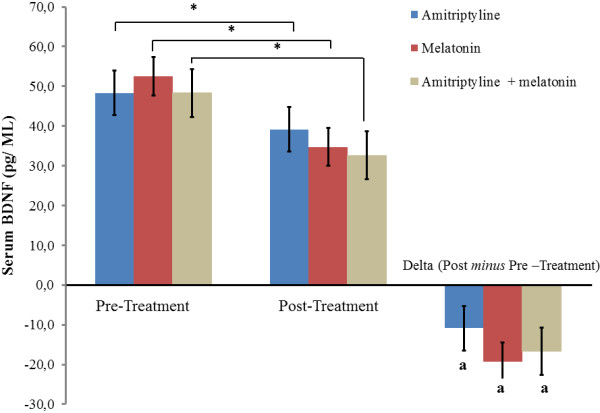
**Mean serum BDNF (ng/mL) at baseline and the after treatment presented as the mean ± SEM.** The asterisk indicates a significant within group difference according to Mixed ANOVA model with Bonferroni test*.* Delta values (serum BDNF before treatment minus serum BDNF after pretreatment) were performed using a Mixed ANOVA*.* The error bars indicate the standard error of the mean. ^a^Indicates that the treatment did not induce an effect that was significantly different between treatment groups (P > 0.05).

Although no significant difference between treatment groups in serum BDNF at baseline was observed, there was large variability in the serum level of this neurotrophin (Table 1). From the baseline, the mean of serum BDNF decreased 22.57% in the amitriptyline group, whereas the melatonin group and the melatonin + amitriptyline group presented a mean reduction of 36.6% and 34.49%, respectively (Figure 4). The effect size within group as assessed by SDM in the amitriptyline group was 0.43 (CI 95%, 0.05-0.83), whereas in the melatonin group, the value was 0.8 (CI 95%, 0.4-1.2), and in the melatonin + amitriptyline group, the value was 0.67 (CI 95%, 0.24-1.09) (Figure 4).

## Discussion

The most clinically relevant finding of this study was melatonin’s ability to improve the function of the inhibitory endogenous pain-modulating system as assessed by the reduction of the NPS_(0–10)_ during the CPM-TASK. In addition, this study highlights that the HPT and BDNF serum levels at baseline were inversely correlated with the magnitude of the treatment effect on the descending modulatory system as assessed by the CPM-TASK. Furthermore, these findings revealed that in general, all treatments improved the FM symptoms (pain, sleep quality); however, the clinical effect was marginally better when the patients used melatonin alone or associated with amitriptyline.

These findings suggest that the melatonin treatment had a direct effect on pain pathways or on the levels of signaling chemicals that regulate pain. Melatonin induced a reduction that was higher than 35% on the NPS_(0–10)_ during the CPM-TASK, and, as can be observed from the lower limit of the confidence interval regarding pain score on the VAS presented as SDM [1.29 (CI 95%, 0.98-1.58)], its effect was larger than that of amitriptyline alone [0.99 (CI 95%, 0.68-1.30)]. The melatonin effect on CPM is biologically plausible, and it is supported by evidence from experimental studies about its effect on GABAergic [48], opioid and glutamatergic systems [49]. In addition, this hypothesis is supported by other studies that have demonstrated a well-characterized anatomical network able to regulate nociceptive processing (largely within the dorsal horn) in various circumstances to produce either facilitation (pronociception) or inhibition (antinociception) [50]. Therefore, melatonin may also inhibit the descending pathways that facilitate pain transmission, which are believed to be circuits with a sustained activation that underlies chronic pain, such as in FM [51,52]. Overall, these findings suggest that melatonin increased the inhibitory descending pain system effect, which involves anatomical connections between cortical and brainstem regions in the human brain, as demonstrated by tractography [53].

The inverse correlation between CPM-TASK, BDNF and HTT0 (Table 2) corroborates the presence of central sensitization theory, which is related to a reduction in the descending inhibitory system activity [54] and to a greater activation in the brainstem [55]. Thus, the reduction of BDNF suggests that this neuromodulator is involved in central sensitization [56,57]. This neurotrophin is involved in altering the excitatory/inhibitory balance in the central nervous system (CNS) and in the amplification of pain response [58,59]. Because BDNF levels can serve as a molecular “sensor” of the global levels of neuronal activity, it has been suggested that the induction of BDNF expression in response to increases in the level of neuronal activity may dampen cortical excitability by promoting the development and/or strengthening of inhibitory synapses in local circuits [60,61]. This multifaceted mechanism of melatonin’s effect on pain also results in a reduction in serum BDNF, an effect observed in this study, which corroborates the results of a previous randomized clinical trial concerning chronic pelvic pain induced by endometriosis [16].

In addition, melatonin’s effect on BDNF observed in this study may be explained by its anti-inflammatory effects. This hypothesis is supported by the relation between BDNF and pro-inflammatory cytokines that has been demonstrated in FM patients [62-64]. Melatonin has marked anti-inflammatory effects on peripheral sites by inhibiting the release of pro-inflammatory cytokines [12]. Additional mechanisms involved in melatonin’s effect on pain pathways include an important reduction of nitric oxide (NO) and malondialdehyde (MDA) levels, two compounds that are closely related to inflammation [65]. Melatonin inhibits the inflammatory response and inducible NO synthase (iNOS) isoform expression [66], as demonstrated in FM patients by Citera et al. [5]. In addition, melatonin as an adjuvant of drugs that increase serotonin (e.g., SSRIs, tricyclic antidepressants) could be a beneficial pharmacological approach for the management of patients with FM [6]. Overall, these findings reveal that melatonin may change a response associated with a maladaptive neuroplasticity process orchestrated by neuronal, endocrinal, and immune mechanisms that can amplify sensory pain signals to the neural pain matrix.

The present study demonstrated that all treatments reduced pain, and in the melatonin-treated groups, this effect was marginally better than in the amitriptyline group. Melatonin’s effect on pain was demonstrated by the VAS, FIQ and PPT. These findings corroborate evidence of experimental studies [8] and previous randomized clinical trials on acute pain [10,14] and chronic pain such as fibromyalgia [5,6], temporomandibular disorders [15], endometriosis [16] and a dose–response study with healthy subjects [26]. The highly lipid-soluble nature of melatonin allows it to easily penetrate the blood–brain barrier. The antinociceptive effect of melatonin is known to involve the activation of supraspinal sites and the inhibition of “spinal windup” [67,68].

Evidence from experimental studies suggests that the analgesic effects of melatonin are mediated by opioids [69] and by gamma-aminobutyric acid ([GABA] ergic) systems [27,28], but it is not possible to dissociate the effect of each individual neurobiological system in human experimental and clinical studies. Indeed, only the net effect can be assessed. Studies have also suggested that additional pathways play a role in the analgesic actions of melatonin, such as the nuclear signaling pathways, receptor-independent radical scavenging, and inhibition of the release of pro-inflammatory cytokines at peripheral sites.

According to previous clinical studies, the most prevalent complaints in patients with FM were sleep disturbance, fatigue, and chronic pain, and these symptoms might be a consequence of melatonin secretion disruption [70]. Moreover, serum levels of melatonin precursors (tryptophan and serotonin) have been reported to be low in patients with FM [71,72]. This could explain the lack of restorative sleep and could be a mechanism involved in dysfunctional pain modulation [3]. Thus, the restoring of melatonin could be an additional mechanism among the others not yet discussed to explain the discrepancy of its effect compared with amitriptyline. However, one can realize that, overall, the association with amitriptyline induced only a marginal increase in the clinical effect. This finding is contrary to our initial hypothesis that the combined treatment (melatonin + amitriptyline) might provide an effect with a higher clinical impact. Although the interventions were randomly allocated to balance the characteristic between arms, some baseline pain traits were higher in group that received melatonin + amitriptyline. This imbalance occurred by chance, but the random allocation ensures the groups were not systematically biased [73]. Furthermore, if any disadvantage would appear, it would be on this group (*i.e.* amitriptyline + melatonin) which had more clinical comorbidities. Accordingly, our findings highlight that melatonin alone or in combination with amitriptyline might improve the efficacy of fibromyalgia treatment in those with more severe symptoms. This finding is in accordance with a recent study that demonstrated an association between lower levels of 6-sulfatoxy-melatonin with higher severe symptoms of FM such as pain, sleep disturbances, fatigue, anxiety and depression [74]. However, to reduce the possible influence of this imbalance in the treatment effect, all analyses were fitted using the delta value of the outcomes from baseline. In addition, these results also agree with previous studies that demonstrate that antidepressant drugs (selective serotonin reuptake inhibitors, SSRIs) associated with melatonin-improved fibromyalgia symptoms [6].

The strengths of this study include the comparison of melatonin and amitriptyline association in a phase II, randomized, double-dummy factorial design, placebo-controlled trial and the use of multiple efficacy and safety measures based on previous trial experience. Although the important effects of SSRIs in FM are well known, the effects of the association of melatonin and amitriptyline in this chronic pain condition were not previously studied. We conducted this trial according to CONSORT guidelines, and given that we used the Delphi List (a list of criteria for the quality assessment of randomized controlled trials), our randomized controlled trial can be considered to be of strong quality because all 8 items in this scale can be positively scored [75]. In addition, we used the double-dummy method and shield placement to prevent the patient and team members from following the patients to control assessment bias. These findings are important because they demonstrate that the application of new therapeutics can be assessed in each particular context to determine whether they provide enough benefit over those already available [76]. The factorial design is a proposed method for examining the interactions between treatments by generating an interaction ratio. In addition, this method permits us to estimate whether the “at the margins” analyses may have overestimated or underestimated the efficacy (and adverse effects) of each agent. A potential limitation is the short treatment duration, but it would have been difficult to justify a prolonged treatment period in patients experiencing chronic pain if they had a high incidence of severe side effects. Accordingly, the incidence of MJSE in the amitriptyline group was 38.09%. This finding is in accordance with the literature reporting that the tricyclic antidepressants are effective at alleviating pain and improving sleep quality [77]; however, the complaints related to common anticholinergic side effects such as dry mouth, sedation, constipation, orthostasis and weight gain are common [78]. We emphasize that the results of this study are relevant only to the patient population investigated. Although the homogeneity of this study population is methodologically advantageous, the issue of external validity arises. Thus, additional research with a larger number of patients is needed to more widely assess the potential benefits of melatonin in several different clinical settings, and future studies are required before definitive conclusions regarding melatonin and pain treatment can be made. Finally, although several strategies were used to prevent patients and the evaluator team from unblinding, formal assessment for awareness of the allocation (either active or placebo) was not performed. However, our objective surrogates less prone to bias (i.e., serum BDNF, analgesics requirements, CPM-TASK) were consistent with pain scores, and thus, unblinding is unlikely to have influenced the direction of our conclusions.

## Conclusion

In conclusion, in this 6-week, randomized, double-dummy, placebo-controlled study, melatonin alone or associated with amitriptyline was better than amitriptyline alone in improving pain on the VAS, FIQ and PPT, whereas its association with amitriptyline produced only marginal additional clinical effects. Melatonin increased the inhibitory endogenous pain-modulating system as assessed by the reduction of the NPS_(0-10)_ during the CPM-TASK. In addition, this study suggests that peripheral BDNF could be used as a biomarker of central sensitization, as it is inversely correlated with pain reduction in the CPM-TASK.

## Abbreviations

PMS: Pain-modulating system; BDNF: Brain-derived neurotrophic factor; HPT: Heat pain threshold; FM: Fibromyalgia; RCT: Randomized clinical trial; NA: Noradrenalin; 5- HT: Serotonin; DA: Dopamine; NO: Nitric oxide; MDA: Malondialdehyde; SSRIs: Selective serotonin reuptake inhibitors; PPT: Pressure pain thresholds; DNIC: Diffuse noxious inhibitory control; CPM: Conditional pain modulation; NPS: Numerical rating pain scale; ELISA: Enzyme-linked immunosorbent assay; NSAIDS: Non-steroidal anti-inflammatory drugs; FIQ: Fibromyalgia impact questionnaire; MSE: Minor side effects; MJSE: Major side effects; lCI: Confidence interval; B- PCS: Pain catastrophizing scale for the brazilian population; VAS: Visual analog scale.

## Competing interests

The authors declare that there are no financial or other relationships that might lead to conflicts of interest involving any of the following arrangements: *financial relationship to the work, employees of a company, consultants for a company, stockholders of the company, members of a speakers bureau or any other form of financial compensation.*

## Author’s contribution

A participated in the sequence alignment. B participated in the design of the study and performed the statistical analysis. C conceived of the study, and participated in its design and coordination and helped to draft the manuscript. All authors read and approved the final manuscript.

## Pre-publication history

The pre-publication history for this paper can be accessed here:

http://www.biomedcentral.com/2050-6511/15/40/prepub

## References

[B1] Mease PJ Clauw DJ Arnold LM Goldenberg DL Witter J Williams DA Simon LS Strand CV Bramson C Martin S Wright TM Littman B Wernicke JF Gendreau RM Crofford LJ Fibromyalgia syndrome J Rheumatol 2005 32 2270 2277 16265715

[B2] Arnold LM Hudson JI Keck PE Auchenbach MB Javaras KN Hess EV Comorbidity of fibromyalgia and psychiatric disorders J Clin Psychiatry 2006 67 1219 1225 16965199 10.4088/jcp.v67n0807

[B3] Wikner J Hirsch U Wetterberg L Röjdmark S Fibromyalgia–a syndrome associated with decreased nocturnal melatonin secretion Clin Endocrinol (Oxf) 1998 49 179 183 9828904 10.1046/j.1365-2265.1998.00503.x

[B4] Bennett RM The rational management of fibromyalgia patients Rheum Dis Clin North Am 2002 28 181 199 v 12122913 10.1016/s0889-857x(02)00002-9

[B5] Citera G Arias MA Maldonado-Cocco JA Lázaro MA Rosemffet MG Brusco LI Scheines EJ Cardinalli DP The effect of melatonin in patients with fibromyalgia: a pilot study Clin Rheumatol 2000 19 9 13 10752492 10.1007/s100670050003

[B6] Hussain SA Al-Khalifa II Jasim NA Gorial FI Adjuvant use of melatonin for treatment of fibromyalgia J Pineal Res 2011 50 267 271 21158908 10.1111/j.1600-079X.2010.00836.x

[B7] Mahdi AA Fatima G Das SK Verma NS Abnormality of circadian rhythm of serum melatonin and other biochemical parameters in fibromyalgia syndrome Indian J Biochem Biophys 2011 48 82 87 21682138

[B8] Laste G Vidor L de Macedo IC Rozisky JR Medeiros L de Souza A Meurer L de Souza IC Torres IL Caumo W Melatonin treatment entrains the rest-activity circadian rhythm in rats with chronic inflammation Chronobiol Int 2013 30 1077 1088 23879696 10.3109/07420528.2013.800088

[B9] Detanico BC Piato AL Freitas JJ Lhullier FL Hidalgo MP Caumo W Elisabetsky E Antidepressant-like effects of melatonin in the mouse chronic mild stress model Eur J Pharmacol 2009 607 121 125 19249298 10.1016/j.ejphar.2009.02.037

[B10] Caumo W Levandovski R Hidalgo MP Preoperative anxiolytic effect of melatonin and clonidine on postoperative pain and morphine consumption in patients undergoing abdominal hysterectomy: a double-blind, randomized, placebo-controlled study J Pain 2009 10 100 108 19010741 10.1016/j.jpain.2008.08.007

[B11] Laste G de Macedo IC Ripoll Rozisky J Ribeiro da Silva F Caumo W Torres IL Melatonin administration reduces inflammatory pain in rats J Pain Res 2012 5 359 362 23204863 10.2147/JPR.S34019 PMC3508662

[B12] Ambriz-Tututi M Granados-Soto V Oral and spinal melatonin reduces tactile allodynia in rats via activation of MT2 and opioid receptors Pain 2007 132 273 280 17346886 10.1016/j.pain.2007.01.025

[B13] Esposito E Cuzzocrea S Antiinflammatory activity of melatonin in central nervous system Curr Neuropharmacol 2010 8 228 242 21358973 10.2174/157015910792246155 PMC3001216

[B14] Caumo W Torres F Moreira NL Auzani JA Monteiro CA Londero G Ribeiro DF Hidalgo MP The clinical impact of preoperative melatonin on postoperative outcomes in patients undergoing abdominal hysterectomy Anesth Analg 2007 105 1263 1271 table of contents 17959953 10.1213/01.ane.0000282834.78456.90

[B15] Vidor LP Torres IL Custódio de Souza IC IC Fregni F Caumo W Analgesic and sedative effects of melatonin in temporomandibular disorders: a double-blind, randomized, parallel-group, placebo-controlled study J Pain Symptom Manage 2013 46 422 432 23195393 10.1016/j.jpainsymman.2012.08.019

[B16] Schwertner A Conceição Dos Santos CC Costa GD Deitos A de Souza A de Souza IC Torres IL da Cunha Filho JS Caumo W Efficacy of melatonin in the treatment of endometriosis: a phase II, randomized, double-blind, placebo-controlled trial Pain 2013 154 874 881 23602498 10.1016/j.pain.2013.02.025

[B17] Yarnitsky D Conditioned pain modulation (the diffuse noxious inhibitory control-like effect): its relevance for acute and chronic pain states Curr Opin Anaesthesiol 2010 23 611 615 20543676 10.1097/ACO.0b013e32833c348b

[B18] Paul-Savoie E Marchand S Morin M Bourgault P Brissette N Rattanavong V Cloutier C Bissonnette A Potvin S Is the deficit in pain inhibition in fibromyalgia influenced by sleep impairments? Open Rheumatol J 2012 6 296 302 23091577 10.2174/1874312901206010296 PMC3474944

[B19] Houvenagel E Forzy G Leloire O Gallois P Hary S Hautecoeur P Convain L Henniaux M Vincent G Dhondt JL Cerebrospinal fluid monoamines in primary fibromyalgia] Rev Rhum Mal Osteoartic 1990 57 21 23 1690912

[B20] Legangneux E Mora JJ Spreux-Varoquaux O Thorin I Herrou M Alvado G Gomeni C Cerebrospinal fluid biogenic amine metabolites, plasma-rich platelet serotonin and [3H] imipramine reuptake in the primary fibromyalgia syndrome Rheumatology (Oxford) 2001 40 290 296 11285376 10.1093/rheumatology/40.3.290

[B21] Russell IJ Vaeroy H Javors M Nyberg F Cerebrospinal fluid biogenic amine metabolites in fibromyalgia/fibrositis syndrome and rheumatoid arthritis Arthritis Rheum 1992 35 550 556 1374252 10.1002/art.1780350509

[B22] Wood PB Glabus MF Simpson R Patterson JC Changes in gray matter density in fibromyalgia: correlation with dopamine metabolism J Pain 2009 10 609 618 19398377 10.1016/j.jpain.2008.12.008

[B23] Potvin S Larouche A Normand E de Souza JB Gaumond I Grignon S Marchand S DRD3 Ser9Gly polymorphism is related to thermal pain perception and modulation in chronic widespread pain patients and healthy controls J Pain 2009 10 969 975 19464960 10.1016/j.jpain.2009.03.013

[B24] Volz MS Medeiros LF Tarragô MG Vidor LP Dall’Agnol L Deitos A Brietzke A Rozisky JR Rispolli B Torres IL Fregni F Caumo W The relationship between cortical excitability and pain catastrophizing in myofascial pain J Pain 2013 14 1140 1147 23810270 10.1016/j.jpain.2013.04.013

[B25] Millan MJ Descending control of pain Prog Neurobiol 2002 66 355 474 12034378 10.1016/s0301-0082(02)00009-6

[B26] Stefani L Muller S Torres I Razzolini B Rozisky J Fregni F Markus R Caumo W A Phase II, randomized, double-blind, placebo controlled, dose–response trial of the melatonin effect on the pain threshold of healthy subjects PLoS One 2013 8 10 e74107.27 10.1371/journal.pone.0074107 PMC3788771 25947930

[B27] Zurowski D Nowak L Machowska A Wordliczek J Thor PJ Exogenous melatonin abolishes mechanical allodynia but not thermal hyperalgesia in neuropathic pain. The role of the opioid system and benzodiazepine-gabaergic mechanism J Physiol Pharmacol 2012 63 641 647 23388480

[B28] Golombek DA Escolar E Burin LJ De Brito Sánchez MG Cardinali DP Time-dependent melatonin analgesia in mice: inhibition by opiate or benzodiazepine antagonism Eur J Pharmacol 1991 194 25 30 2060591 10.1016/0014-2999(91)90119-b

[B29] Schulz KF Altman DG Moher D Group C CONSORT 2010 statement: updated guidelines for reporting parallel group randomised trials Int J Surg 2011 9 672 677 22019563 10.1016/j.ijsu.2011.09.004

[B30] Wolfe F Clauw DJ Fitzcharles MA Goldenberg DL Katz RS Mease P Russell AS Russell IJ Winfield JB Yunus MB The American College of Rheumatology preliminary diagnostic criteria for fibromyalgia and measurement of symptom severity Arthritis Care Res (Hoboken) 2010 62 600 610 20461783 10.1002/acr.20140

[B31] Scott J Huskisson EC Graphic representation of pain Pain 1976 2 175 184 1026900

[B32] Couto C de Souza IC Torres IL Fregni F Caumo W Paraspinal stimulation combined with trigger point needling and needle rotation for the treatment of myofascial pain: a randomized sham-controlled clinical trial Clin J Pain 2014 30 214 223 23629597 10.1097/AJP.0b013e3182934b8d

[B33] Dao TT Lavigne GJ Feine JS Tanguay R Lund JP Power and sample size calculations for clinical trials of myofascial pain of jaw muscles J Dent Res 1991 70 118 122 1991868 10.1177/00220345910700020401

[B34] Kaipper MB Chachamovich E Hidalgo MP Torres IL Caumo W Evaluation of the structure of Brazilian State-Trait Anxiety Inventory using a Rasch psychometric approach J Psychosom Res 2010 68 223 233 20159207 10.1016/j.jpsychores.2009.09.013

[B35] Hamilton M A rating scale for depression J Neurol Neurosurg Psychiatry 1960 23 56 62 14399272 10.1136/jnnp.23.1.56 PMC495331

[B36] Buysse DJ Reynolds CF 3rd Monk TH Berman SR Kupfer DJ The Pittsburgh Sleep Quality Index: a new instrument for psychiatric practice and research Psychiatry Res 1989 28 193 213 2748771 10.1016/0165-1781(89)90047-4

[B37] Lobbestael J Leurgans M Arntz A Inter-rater reliability of the Structured Clinical Interview for DSM-IV Axis I Disorders (SCID I) and Axis II Disorders (SCID II) Clin Psychol Psychother 2011 18 75 79 20309842 10.1002/cpp.693

[B38] Sehn F Chachamovich E Vidor LP Dall-Agnol L de Souza IC Torres IL Fregni F Caumo W Cross-cultural adaptation and validation of the Brazilian Portuguese version of the pain catastrophizing scale Pain Med 2012 13 1425 1435 23036076 10.1111/j.1526-4637.2012.01492.x

[B39] Burckhardt CS Clark SR Bennett RM The fibromyalgia impact questionnaire: development and validation J Rheumatol 1991 18 728 733 1865419

[B40] Marques A Santos A Assumpcao A Matsutani L Lage L Pereira C Validation of a Brazilian version of the Fibromyalgia Impact Questionnaire (FIQ) Ann Rheum Dis 2006 65 557 55716531558

[B41] Fischer AA Pressure algometry over normal muscles. Standard values, validity and reproducibility of pressure threshold Pain 1987 30 115 126 3614975 10.1016/0304-3959(87)90089-3

[B42] Tousignant-Laflamme Y Pagé S Goffaux P Marchand S An experimental model to measure excitatory and inhibitory pain mechanisms in humans Brain Res 2008 1230 73 79 18652808 10.1016/j.brainres.2008.06.120

[B43] Magerl W Krumova EK Baron R Tölle T Treede RD Maier C Reference data for quantitative sensory testing (QST): refined stratification for age and a novel method for statistical comparison of group data Pain 2010 151 598 605 20965658 10.1016/j.pain.2010.07.026

[B44] Marchand S Arsenault P Spatial summation for pain perception: interaction of inhibitory and excitatory mechanisms Pain 2002 95 201 206 11839419 10.1016/S0304-3959(01)00399-2

[B45] Schestatsky P Valls-Solé J Costa J León L Veciana M Chaves ML Skin autonomic reactivity to thermoalgesic stimuli Clin Auton Res 2007 17 349 355 18049833 10.1007/s10286-007-0446-8

[B46] Tesarz J Gerhardt A Schommer K Treede RD Eich W Alterations in endogenous pain modulation in endurance athletes: an experimental study using quantitative sensory testing and the cold-pressor task Pain 2013 154 1022 1029 23657118 10.1016/j.pain.2013.03.014

[B47] Kazis LE Anderson JJ Meenan RF Effect sizes for interpreting changes in health status Med Care 1989 27 S178 S189 2646488 10.1097/00005650-198903001-00015

[B48] Wilhelmsen M Amirian I Reiter RJ Rosenberg J Gögenur I Analgesic effects of melatonin: a review of current evidence from experimental and clinical studies J Pineal Res 2011 51 270 277 21615490 10.1111/j.1600-079X.2011.00895.x

[B49] Mantovani M Kaster MP Pertile R Calixto JB Rodrigues AL Santos AR Mechanisms involved in the antinociception caused by melatonin in mice J Pineal Res 2006 41 382 389 17014696 10.1111/j.1600-079X.2006.00380.x

[B50] Basbaum AI Fields HL Endogenous pain control systems: brainstem spinal pathways and endorphin circuitry Annu Rev Neurosci 1984 7 309 338 6143527 10.1146/annurev.ne.07.030184.001521

[B51] Gebhart GF Descending modulation of pain Neurosci Biobehav Rev 2004 27 729 737 15019423 10.1016/j.neubiorev.2003.11.008

[B52] Porreca F Ossipov MH Gebhart GF Chronic pain and medullary descending facilitation Trends Neurosci 2002 25 319 325 12086751 10.1016/s0166-2236(02)02157-4

[B53] Hadjipavlou G Dunckley P Behrens TE Tracey I Determining anatomical connectivities between cortical and brainstem pain processing regions in humans: a diffusion tensor imaging study in healthy controls Pain 2006 123 169 178 16616418 10.1016/j.pain.2006.02.027

[B54] Schwenkreis P Janssen F Rommel O Pleger B Völker B Hosbach I Dertwinkel R Maier C Tegenthoff M Bilateral motor cortex disinhibition in complex regional pain syndrome (CRPS) type I of the hand Neurology 2003 61 515 519 12939426 10.1212/wnl.61.4.515

[B55] Graven-Nielsen T Wodehouse T Langford RM Arendt-Nielsen L Kidd BL Normalization of widespread hyperesthesia and facilitated spatial summation of deep-tissue pain in knee osteoarthritis patients after knee replacement Arthritis Rheum 2012 64 2907 2916 22421811 10.1002/art.34466

[B56] Delafoy L Gelot A Ardid D Eschalier A Bertrand C Doherty AM Diop L Interactive involvement of brain derived neurotrophic factor, nerve growth factor, and calcitonin gene related peptide in colonic hypersensitivity in the rat Gut 2006 55 940 945 16401692 10.1136/gut.2005.064063 PMC1856334

[B57] Groth R Aanonsen L Spinal brain-derived neurotrophic factor (BDNF) produces hyperalgesia in normal mice while antisense directed against either BDNF or trkB, prevent inflammation-induced hyperalgesia Pain 2002 100 171 181 12435470 10.1016/s0304-3959(02)00264-6

[B58] Sommer C Kress M Recent findings on how proinflammatory cytokines cause pain: peripheral mechanisms in inflammatory and neuropathic hyperalgesia Neurosci Lett 2004 361 184 187 15135924 10.1016/j.neulet.2003.12.007

[B59] Kerr BJ Bradbury EJ Bennett DL Trivedi PM Dassan P French J Shelton DB McMahon SB Thompson SW Brain-derived neurotrophic factor modulates nociceptive sensory inputs and NMDA-evoked responses in the rat spinal cord J Neurosci 1999 19 5138 5148 10366647 10.1523/JNEUROSCI.19-12-05138.1999 PMC6782650

[B60] Genoud C Knott GW Sakata K Lu B Welker E Altered synapse formation in the adult somatosensory cortex of brain-derived neurotrophic factor heterozygote mice J Neurosci 2004 24 2394 2400 15014114 10.1523/JNEUROSCI.4040-03.2004 PMC6729494

[B61] Rutherford LC DeWan A Lauer HM Turrigiano GG Brain-derived neurotrophic factor mediates the activity-dependent regulation of inhibition in neocortical cultures J Neurosci 1997 17 4527 4535 9169513 10.1523/JNEUROSCI.17-12-04527.1997 PMC6573348

[B62] Ortega E García JJ Bote ME Martín-Cordero L Escalante Y Saavedra JM Northoff H Giraldo E Exercise in fibromyalgia and related inflammatory disorders: known effects and unknown chances Exerc Immunol Rev 2009 15 42 65 19957871

[B63] Bote ME García JJ Hinchado MD Ortega E Inflammatory/stress feedback dysregulation in women with fibromyalgia Neuroimmunomodulation 2012 19 343 351 22986514 10.1159/000341664

[B64] Wallace DJ Linker-Israeli M Hallegua D Silverman S Silver D Weisman MH Cytokines play an aetiopathogenetic role in fibromyalgia: a hypothesis and pilot study Rheumatology (Oxford) 2001 40 743 749 11477278 10.1093/rheumatology/40.7.743

[B65] Bilici D Akpinar E Kiziltunç A Protective effect of melatonin in carrageenan-induced acute local inflammation Pharmacol Res 2002 46 133 139 12220952 10.1016/s1043-6618(02)00089-0

[B66] Cuzzocrea S Zingarelli B Gilad E Hake P Salzman AL Szabó C Protective effect of melatonin in carrageenan-induced models of local inflammation: relationship to its inhibitory effect on nitric oxide production and its peroxynitrite scavenging activity J Pineal Res 1997 23 106 116 9392449 10.1111/j.1600-079x.1997.tb00342.x

[B67] Morgan P Barrett P Howell H Helliwell R Melatonin receptors - localization, molecular pharmacology and physiological significance Neurochem Int 1994 24 101 146 8161940 10.1016/0197-0186(94)90100-7

[B68] Noseda R Hernández A Valladares L Mondaca M Laurido C Soto-Moyano R Melatonin-induced inhibition of spinal cord synaptic potentiation in rats is MT2 receptor-dependent Neurosci Lett 2004 360 41 44 15082174 10.1016/j.neulet.2004.01.080

[B69] Li SR Wang T Wang R Dai X Chen Q Li RD Melatonin enhances antinociceptive effects of delta-, but not mu-opioid agonist in mice Brain Res 2005 1043 132 138 15862526 10.1016/j.brainres.2005.02.067

[B70] Bazzichi L Rossi A Giacomelli C Bombardieri S Exploring the abyss of fibromyalgia biomarkers Clin Exp Rheumatol 2010 28 S125 S130 21176432

[B71] Yunus MB Fibromyalgia and overlapping disorders: the unifying concept of central sensitivity syndromes Semin Arthritis Rheum 2007 36 339 356 17350675 10.1016/j.semarthrit.2006.12.009

[B72] Yunus MB Role of central sensitization in symptoms beyond muscle pain, and the evaluation of a patient with widespread pain Best Pract Res Clin Rheumatol 2007 21 481 497 17602995 10.1016/j.berh.2007.03.006

[B73] Fives AW Russell D Kearns N Rena Lyons G Eaton P Canavan J Devaney C O’Brien A The role of random allocation in randomized controlled trials: distinguishing selection bias from baseline imbalance J Multidisciplinary Eval 2013 9 33 42

[B74] Pernambuco AP Schetino LP Viana RS Carvalho LS Reis D The involvement of melatonin in the clinical status of patients with fibromyalgia syndrome Clin Exp Rheumatol 2014 in press in press 24565062

[B75] Verhagen AP de Vet HC de Bie RA Kessels AG Boers M Bouter LM Knipschild PG The Delphi list: a criteria list for quality assessment of randomized clinical trials for conducting systematic reviews developed by Delphi consensus J Clin Epidemiol 1998 51 1235 1241 10086815 10.1016/s0895-4356(98)00131-0

[B76] Cazzola M Application of Number Needed to Treat (NNT) as a Measure of Treatment Effect in Respiratory Medicine Treat Respir Med 2006 5 79 84 16512688 10.2165/00151829-200605020-00001

[B77] Plesh O Curtis D Levine J McCall WD Amitriptyline treatment of chronic pain in patients with temporomandibular disorders J Oral Rehabil 2000 27 834 841 11065017 10.1046/j.1365-2842.2000.00572.x

[B78] Bendtsen L Jensen R Amitriptyline reduces myofascial tenderness in patients with chronic tension-type headache Cephalalgia 2000 20 603 610 11075846 10.1046/j.1468-2982.2000.00087.x

